# Cardiac Implantable Electronic Device Educational Application for Cardiac Anesthesiology Trainees: Tutorial on App Development

**DOI:** 10.2196/60087

**Published:** 2025-07-29

**Authors:** Ahmed Zaky, Aisha Waheed, Brittany Hatter, Srilakshmi Malempati, Sai Hemanth Maremalla, Ragib Hasan, Yuliang Zheng, Scott Snyder

**Affiliations:** 1 Department of Anesthesiology and Perioperative Medicine, University of Alabama at Birmingham, 619 South 19th Street, JT 804, Birmingham, AL, 35249-6810, United States, 1 3522176319; 2 Department of Internal Medicine, Princeton Baptist Health System, Birmingham, AL, United States; 3 Department of Computer Sciences, University of Alabama at Birmingham, Birmingham, AL, United States; 4 Department of Education, University of Alabama at Birmingham, Birmingham, AL, United States

**Keywords:** pacemakers, curriculum, mobile, education, rhythm devices, anesthesiology trainees, technology, medical education, cardiac, cardiac implantable, electronic device, application, app, anesthesiology, cardiothoracic anesthesiology, trainee, assessment

## Abstract

Despite the exposure of cardiothoracic anesthesiology trainees to patients with cardiac implantable electronic devices (CIEDs), there is a paucity of formal curricula on this subject. Major impediments to educating cardiothoracic anesthesiology trainees on CIEDs include busy clinical schedules, short staffing, inconsistent trainees’ exposure to CIEDs, multiplicity of vendors, and a “millennial” mentality of the new generation of learners. As a result, cardiothoracic anesthesiology trainees graduating from their residency and fellowship programs may lack the competency to manage patients with CIEDs. Herein, we report our systematic approach to designing, validating, mapping, evaluating, and delivering a CIED curriculum on the first mobile app of its kind on this subject. Development of the CIED curriculum proceeded through the Kern 6-step approach of problem identification, determining and prioritizing content, writing goals and objectives, selecting instructional strategies, implementation of the material, and evaluation and applications of lessons learned. This was followed by the delivery of the curriculum in the form of a user-study app and administrator-type app with functionalities in the assessment of the learners’ gains, experience, and satisfaction as well as the administrator’s capability to update the educational content based on the feedback of the learners and the emerging technology. As such, the CIED app allows asynchronous learning at the pace of the learners and allows, through a multiplicity of educational materials, the ability to digest this complex and understudied subject. We report on the pilot phase of the project. We benefit from the experience of a multidisciplinary team of anesthesiologists, computer scientists, and educators in accomplishing this project.

## Introduction

Cardiac implantable electronic devices (CIEDs) are electronic devices placed in patients experiencing permanent life-threatening arrhythmias. They include pacemakers, implantable cardioverter-defibrillators (ICD), and cardiac resynchronization therapies (CRT). The number of CIEDs implanted is increasing rapidly worldwide [1]. Due to increased longevity and technological advancement, the number of patients with CIEDs undergoing surgical procedures is steadily increasing [2]. With a growing sophistication of the technology and the potential for preventable adverse events related to CIED dysfunctionality in the perioperative period [3], there is a need for anesthesiology trainees to gain the knowledge and skills necessary to manage CIEDs perioperatively [4].

Despite an increased exposure of anesthesiology trainees to patients with CIEDs, and the availability of anesthesiology-led CIED services [5], there have not been formal curricula to educate trainees on this complex subject. Furthermore, busy clinical schedules, staff shortages, relatively short duration of the cardiothoracic rotation, the unpredicted and inconsistent exposure of trainees to patients with CIEDs, and the “millennial” mentality of the current generation of learners [6], all have challenged synchronous teaching and learning modalities. As a consequence, currently graduating anesthesiology trainees may lack the competency needed to manage patients with CIEDs in their future careers.

Mobile learning (m-learning) is a learning mode that uses a mobile device or tablet. It allows self-directed asynchronous learning at the pace of the learner and hence frees the learner of time and physical constraints. M-learning was shown to be associated with higher learning gains compared with conventional learning in a multitude of medical specialty education [7-10]. At the time of writing this study, there are only 4 programs in the country where CIEDs are perioperatively managed by anesthesiologists with inconsistencies in the educational methodologies delivered to the trainees on this subject.

To remedy the current gap, we describe in this tutorial the systematic process of development and delivery of a high-quality CIED educational curriculum through a mobile app platform. This manuscript should be of interest to anesthesiology educators seeking to incorporate new competencies and rubrics related to CIED into residency training programs.

## Methods

### Development of High-Quality CIED Educational Curriculum

Using the Kern 6-step approach to curriculum development [11], we created goals, learning objectives, instructional content, and pre and postassessments to be delivered via m-learning, and user feedback regarding CIEDs. The Kern framework for curriculum development has been applied for training across multiple medical specialties [12-14]. The authors expanded the 6 steps by including an expert review of the instructional objectives.

Due to the perioperative nature of CIEDs, the curriculum focuses on knowledge and skills within the Accredited Council of Graduate Medical Education (ACGME) domain of Patient Care.

The basic process of curriculum development in medical education has been outlined [12-14] as consisting of the following steps:

1. Problem identification
2. Determining and prioritizing content (focusing on the needs assessment).
3. Writing goals and objectives.
4. Selecting instructional strategies.
5. Implementation of the curriculum.

Evaluation and application of lessons learned.

#### Problem Identification

Several intersecting challenges motivated the development of a curriculum for CIED management. As indicated earlier, technological advances and increased life expectancy have resulted in a steady increase in the number of CIEDs implanted annually [2]. Similarly, there has been an increase in the number of patients with CIEDs undergoing surgical procedures. Due to the rates of postoperative CIED-related complications, CIED-related deaths, and hospital readmissions related to CIEDs, there is a specific need for training in the knowledge and skills needed to manage CIEDs in the perioperative period. Cardiothoracic anesthesiology trainees are extensively exposed to patients with CIEDs during their training. Training ranges from a month-long rotation in the cardiac operating rooms and cardiac surgical intensive care units as an elective or core rotation during residency to a 12-month dedicated accredited clinical fellowship. Despite evidence that the initiation of perioperative anesthesiology-based CIED service is associated with better patient outcomes [5,15-17], there are no standardized published training curricula regarding CIEDs for cardiothoracic anesthesiology trainees. While these conditions highlight the general need for a curriculum focused on CIED interrogation for cardiothoracic anesthesiology trainees and fellows, a specific problem prompted the efforts of this project. A problem involving case delays of procedures on patients with CIEDs due to the show of device representatives and cardiology fellows and the occurrence of multiple preventable patient safety events due to perioperative CIED malfunctions triggered our institution to seek the initiation of an anesthesiologists-led CIED service. A dual-trained cardiothoracic and critical care anesthesiologist with expertise and training in CIED management (AZ) championed this quality and safety initiative. The implementation of an anesthesiologist-led CIED service created an in-parallel need to train anesthesiology trainees with a similar scope of practice to sustain the service. This need was based on an average number of 400 patients with CIED undergoing cardiac and noncardiac procedures at our institution per year. We reasoned that the rising use and exposure to CIED, the complexities of perioperative management of these devices, and the clear gap resulting from the absence of a standard methodology for educating cardiothoracic anesthesiology trainees all justify the design and delivery of a high-quality CIED curriculum for these trainees.

#### Determining and Prioritizing Content (Needs Assessment)

We conducted a needs assessment to assess the current status of CIED education to cardiothoracic anesthesiology trainees in our institution and nationwide and determine potential areas for improvement. Our needs assessment focused on 3 aspects. First, scanning the literature and anesthesiology program websites on published educational material on CIEDs for trainees. Second, we conducted a survey via Qualtrics to cardiothoracic anesthesiology trainees at our institution and at the Ochsner Clinic. The survey included questions about the level of training, the form of CIED didactics, the fallbacks of the current teaching, and what the trainees would like to see in a CIED curriculum. The third aspect of the needs assessment was in the form of in-person informal interviews with residents and cardiothoracic anesthesia trainees at our institution. Interviewees were asked open-ended questions about the current teaching of CIEDs at our institution and areas for improvement. A total of 6 interviews were conducted, 10‐15 minutes each, with 2 trainees at a time. The interviews took place during breaks from working hours in the anesthesia lounge. The PI took notes and observed the reactions of the interviewees during the interviews. The PI then presented the results of the needs assessment to the Cardiothoracic Fellowship Program Director and the Residency Program Director at our institution. The results of the needs assessment are summarized in Textbox 1.

Textbox 1. The results of our needs assessment are summarized as follows:

- At the time of writing, there were 4 institutions that deliver formal cardiac implantable electronic device (CIED) didactics to cardiothoracic anesthesia trainees and fellows.
- Didactics are in the form of website videos (2 institutions) and scheduled lectures (3 institutions).
- Didactic materials were in the form of textbooks, review articles, and on-site workshops at national societal meetings on a yearly basis.
- Most of the didactics are directed to the simpler programming of temporary epicardial pacers that are placed intraoperatively. As a consequence, there is no formal interrogation of these devices [18].
- Trainees at our institution had not received instructional material about CIEDs before the interviews and survey dates.
- Lecture-based training was the predominant instructional approach trainees experienced and was also the least desirable.
- Approximately one-third of trainees rated app-based instruction as their first or second choice of instructional method (hands-on was the most desirable approach).
- Almost three-fourths of respondents indicated that they would prefer a self-paced e-learning system for learning about CIEDs.

The collective results of our needs assessment led us to reason a need to test the design, development, and delivery of a formal comprehensive CIED curriculum to our cardiothoracic anesthesia trainees that adapts to their time constraints.

#### Conceptual Framework

Conceptual frameworks represent ways of thinking about a problem and responding to the complexity of an educational phenomenon. Adding a conceptual framework to best practice approaches such as the Kern 6 step-approach for curriculum design helps construct goals and objectives. Furthermore, this integration helps select the educational intervention to achieve the educational goals and objectives and determine the evaluative methodology of the educational intervention, which will eventually determine the outcomes to be assessed and the evaluative strategy to determine the success of these outcomes [19]. Overall, both best practice and conceptual framework contribute to the rigor of educational scholarship.

Around 2 theories contributed to the design and development of the CIED curriculum, based on the needs assessment. The self-regulated framework, attributed to Zimmerman and Schunk, entails that learners plan, monitor, and evaluate their own learning to achieve their goals [20]. The self-regulated theory is applicable to the busy schedule of cardiothoracic anesthesiology trainees that interferes with synchronous learning, and importantly, to the maturity level of trainees at this stage of their career. The self-regulated theory thus calls for the design of an asynchronous learning method in the form of m-learning. The self-regulated framework was used to formulate the research question: whether an app-delivered CIED curriculum is a suitable delivery method for cardiothoracic anesthesiology trainees.

The Kolb experiential learning cycle that describes the 4-stage learning cycle in the form of concrete experience, reflective observation, abstract conceptualization, and active experimentation is another framework that influenced the design of the practical modules of the curriculum [21]. The framework guided the formulating of the research question: whether video recording of the interrogation and programming procedures of CIEDs by vendor will allow trainees to gain the skills needed to perform these procedures through Kolb stage of reflective observation?

#### Establishing Goals and Objectives

Based on a review of textbooks and consultation with colleagues, the first author developed an initial draft of seventeen major goals and associated instructional objectives (5‐15 objectives for each goal). Overall, curricular goals focused on knowledge, psychomotor skills, and attitudes of the learners and were tailored in a stepwise fashion from acquiring foundational knowledge to applying this knowledge to acquiring the skills in managing CIEDs. For example, the curriculum progresses from understanding and recognizing electrocardiographic patterns of pertinent tachyarrhythmias and bradyarrhythmias to understanding the basics of CIED indications, structure, and function of CIEDs, to recognizing pacer rhythms and understanding the 4 basic parameters used in interrogating and programming CIEDs on different vendors. The final modules of the curriculum are in the form of recorded videos of the performance of device interrogation and programming on the 4 contemporary device vendors. Assessment of the psychomotor skills of the trainees will be in the form of a video recording of the trainee conducting the 4 steps of device programming and interrogations in the form of (1) identification of the vendor, (2) identification of the device, (3) assessment of pacing dependency, and (4) performance of battery and leads functionality in the form of battery longevity, leads impedances, and sensing amplitude and capture threshold. An assessment rubric was created to grade the performer’s skill (Multimedia Appendix 1).

Following the development of 17 goals and objectives associated with such goals, we sought to evaluate the proposed goals and objectives by surveying a group of 17 CIED subject matter experts (SME) on whether (1) the objectives are critical or important to CA learners, (2) the instructional objectives fit the learning goals, and (3) whether the objectives as written were clear. After the initial development of the goals and objectives, the process to evaluate the objectives was similar to methods used by a previous medical curriculum validation process used in obstetrics in Canada [22].

The curriculum validation process involved the identification of a national panel of experts in curricula relating to CIEDs. The definition of expertise of an expert was based on the qualifications: board certification in Cardiology, Electrophysiology, and equivalent certification in CIEDs with greater than 5 years of experience, and an expertise in medical curricular design.

Each panelist received an online survey (Qualtrics) which presented the 17 major learning goals of the curriculum and the instructional objectives for each goal (5‐15 objectives per goal). Respondents were asked to rate the importance of each instructional objective (not important, important, and essential), the fit of the objective to the learning goal (does not fit and aligns), and the clarity of the objective (unclear or ambiguous and clear).

### Ethical Considerations

The study was approved by the University of Alabama Institutional Review Board (IRB00012550) and adheres to the applicable CONSORT (Consolidated Standards of Reporting Trials) guidelines. The requirement for patient consent was waived.

## Results

### Response to Validation of Goals and Objectives

A total of 17 SMEs responded to the Qualtrics survey. The results of the validation survey are in the process of publication elsewhere. In brief, all objectives were rated as important or essential by 80% or more of the 17 raters, several goals had fewer than 70% of objectives rated as essential by 73% or more of the experts, with 6 goals having no objective that was rated as “essential” by more than 73% of experts. A goal of understanding the unique features of individual CIED vendors received the lowest rating given that this subject was not pertinent to cardiothoracic anesthesiologists.

In response to SMEs’ response, we have kept objectives that were either essential or important to greater than 75% of the raters and removed the objectives that were considered important by less than 75% of raters. Furthermore, goals whose objectives were considered important by less than 75% of the raters were removed.

The modified curriculum was then named “Anesthesiology Prospective-basic Curriculum” (Textbox 2).

Textbox 2. **Basic cardiac implantable electronic device (CIED) curriculum: anesthesiology perspective**

Module 1: Basic cardiac electrophysiology
  1A. Basics of surface electrocardiogram
  1B. Heart blocks
  1C. Pertinent tachyarrhythmias
Module 2: Indications of pacemakers, implantable cardioverter defibrillators (ICDs), and cardiac resynchronization therapies (CRTs)
  2A. Permanent pacemaker (PPM) indications
  2B. ICD indications
  2C. CRT indications
Module 3: Anatomy of pacemakers
  3A. Generators
  3B. Leads
Module 4: Pacemaker modes and codes
  4A. Generic pacemaker codes and modes
  4B. Ventricle paced, Ventricle sensed, Inhibited response (VVI) pacing as an example of single chamber pacing
  4C. Dual chamber paced, Dual chamber sensed, Dual response (DDD) pacing as an example of dual chamber pacing
Module 5: Anatomy of ICDs
Module 6: Operation of ICDs
  6A. ICD recognition of arrhythmias
  6B. ICD treatment of arrhythmias
Module 7: Guidelines for perioperative management of CIEDs
Module 8: Magnet behavior
Module 9: Programming and interrogation of CIEDs
Module 10: University of Alabama (UAB) Anesthesia Clinical Protocol of Perioperative CIED Management

### Selection of Instructional Modality and Strategies

One focus of this curriculum development project was the commitment to delivering instructional content and resources, assessment, and program evaluation (eg, usage, acceptability, and satisfaction) of the curriculum via an updatable, accessible, asynchronous electronic platform. An m-learning platform for delivering the curriculum was considered essential for accommodating the constraints experienced by cardiothoracic anesthesiology trainees. Delivering the curriculum via modular delivery of content provides a flexible, self-paced, competency-focused approach to instruction and assessment.

In order to develop the m-learning platform within a structure that would be manageable to learners, the retained goals and objectives were distributed among 11 modules and submodules. The use of modules is a common structure for m-learning platforms [23,24]. Curricular mapping between goals, objectives, the instructional resources associated with each objective, and the assessment for each objective was created for all goals and objectives within the curriculum (Table 1).

**Table 1.** Instructional map for module 1A, 1B, and 1C

Module	Goals	Objective	Instruction	Assessment
1A	Understand basics of heart rhythm formation and propagation Establish a foundation for recognizing paced rhythms	Differentiate between pacemaker and myocardial cells Differentiate the action potential for both pacemaker and myocardial cells Understand the ionic basis responsible for different phases of action potentials Recognize and define refractory periods on an action potential	Text Differentiate between pacemaker cells and myocardial cells	Written quiz with visuals
1B	Understand basics of electrocardiogram depiction Understand the various types of heart block	Determine heart rates on EKG by at least 2 methods Identify the axis on surface EKG Identify different types and levels of heart block on surface EKG	Visuals Text	Written quiz with visuals
1C	Recognize the different types of supraventricular tachycardias Understand the differences between ventricular and supraventricular tachycardias	Describe EKG patterns of SVT Determine electrocardiogram pattern of atrial fibrillation Describe electrocardiogram patterns of AVRNTa Describe electrocardiogram patterns of atrial tachycardias Describe electrocardiogram pattern of atrial flutter Recognize EKG pattern of VT Monomorphic Polymorphic VF Accelerated idioventricular rhythm Understand common SA node abnormalities Describe wide complex SVT Differentiate wide complex SVT from VT	Visuals Text	Written quiz with visuals

Assessment materials were developed in the form of pre and postinstructional quizzes. Postinstructional quizzes were slightly different in order and content from prequizzes. The rationale for this difference is to assess trainees’ incremental learning gains after reading the modular instructional material and to assess whether the postquiz responses were due to knowledge acquisition versus memorization of prequiz grading of wrong answers. A minimum passing score of 80% was used as the criterion for passing any quiz. Quizzes consisted of both multiple-choice questions (MCQ) and open-ended questions. The former were created to assess knowledge, comprehension, and simple applications, while the latter were created to assess a deeper level of learning and ability to explain the reasoning behind the responses [25]. Grading of open-ended questions was performed by the instructor, while grading for MCQs was performed automatically. Quizzes could be taken indefinitely until passing scores are achieved. Assessment for modules that covered skills was in the form of virtual actual device interrogations. This was in the form of performing 4 basic steps: identification of CIED vendor, determination of the type of CIED, determination of pacer dependency, and determination of device functionality in terms of battery longevity, leads’ function in terms of sensing and capture thresholds, and leads’ impedances. Programming entailed adjusting the device settings to suit the site of the surgical procedure and the use of electrosurgical interference, applying a magnet when indicated, and disabling tachycardia therapy in patients with implantable cardioverter defibrillators. Device restoration included restoring of basic settings of the device after the completion of the procedure and enabling tachycardia therapies for ICDs. The interrogations, programming, and restoration were performed under the supervision of the PI on the protocol.

The CIED curriculum underwent an instructional and content validity assessment as a final evaluation step. The instructional validity is aimed at engaging a cohort of SEM to judge whether the instructional content and resources within the app provide a valid representation of each instructional objective. The content validity assessment tested whether the pre-post quizzes were adequately informed by the instructional content and resources linked to the objective. The rationale for engaging SMEs on instructional and content validity is to consolidate the validation of the alignment of goals to objectives. Engagement was in the form of the Qualtrics survey sent to the same SMEs who were provided with the assessment and instructional and assessment material via email. Both the instructional and content validity assessment provided validation of the curricular map in the form of alignment of goals to objectives and to instructional material to assessment. This validation process is conceptually distinct from the assessment of learners’ satisfaction of the curricular content, which is in the form of a short survey embedded at the end of each module. The validation process in this form is consistent with the Accreditation Council of Graduate Medical Education (ACGME) [26-28] (Table 2).

**Table 2.** Alignment of curricular contents with Accreditation Council of Graduate Medical Education core competencies.

ACGME^a^ core competency	Corresponding curricular material to achieve competency
Patient care: “To demonstrate compassionate, appropriate, and effective care for patients, including the ability to perform comprehensive history and physical examinations, formulate diagnostic plans, and coordinate patient care.”	History and physical examination of the patient to determine pertinent information about the CIED^b^ (indications, type, vendor, previous interrogation reports).
Medical Knowledge: “To possess a strong foundation of biomedical knowledge and the ability to apply it to patient care, staying current with evolving medical knowledge.”	Instructional material in the form of module narrative, constantly updated narratives in response to new guidelines and latest technology (leadless pacemakers and subcutaneous ICDs)^c^, library of updated articles
Practice-based learning: “to investigate and evaluate their own care, appraise scientific evidence, and continuously improve their practice through self-evaluation and lifelong learning.”	Unlimited attempts at solving the quizzes, interactive platform between the user study app and the admin app to allow interactive feedback from instructor to learner on performance
Interpersonal and communication skills: “to effectively communicate with patients, families, and other healthcare professionals, building strong relationships and fostering trust.”	Adequate documentation of the interrogation and programming procedure, communication with surgical and EP services on device malfunction issues in need of intervention.
Professionalism: “To demonstrate ethical conduct, respect for colleagues, and a commitment to patient well-being, upholding the highest standards of professional behavior.”	Competency adequately emphasized throughout the instructional material and skill assessment of the curriculum.
Systems-based practice: “To understand and navigate healthcare systems, working effectively within teams and advocating for patients’ needs within the context of the healthcare system.”	Detailed instruction on navigating EMRs^d^, checklists on consulting other services and contacting device reps for vendor-specific issues.

a ACGME: Accreditation Council of Graduate Medical Education.

b CIED: cardiac implantable electronic devices.

c ICD: implantable cardioverters defibrillators.

d EMR: electronic medical records.

### Delivery of the Curriculum

#### Pilot Phase

**Figure 1.**
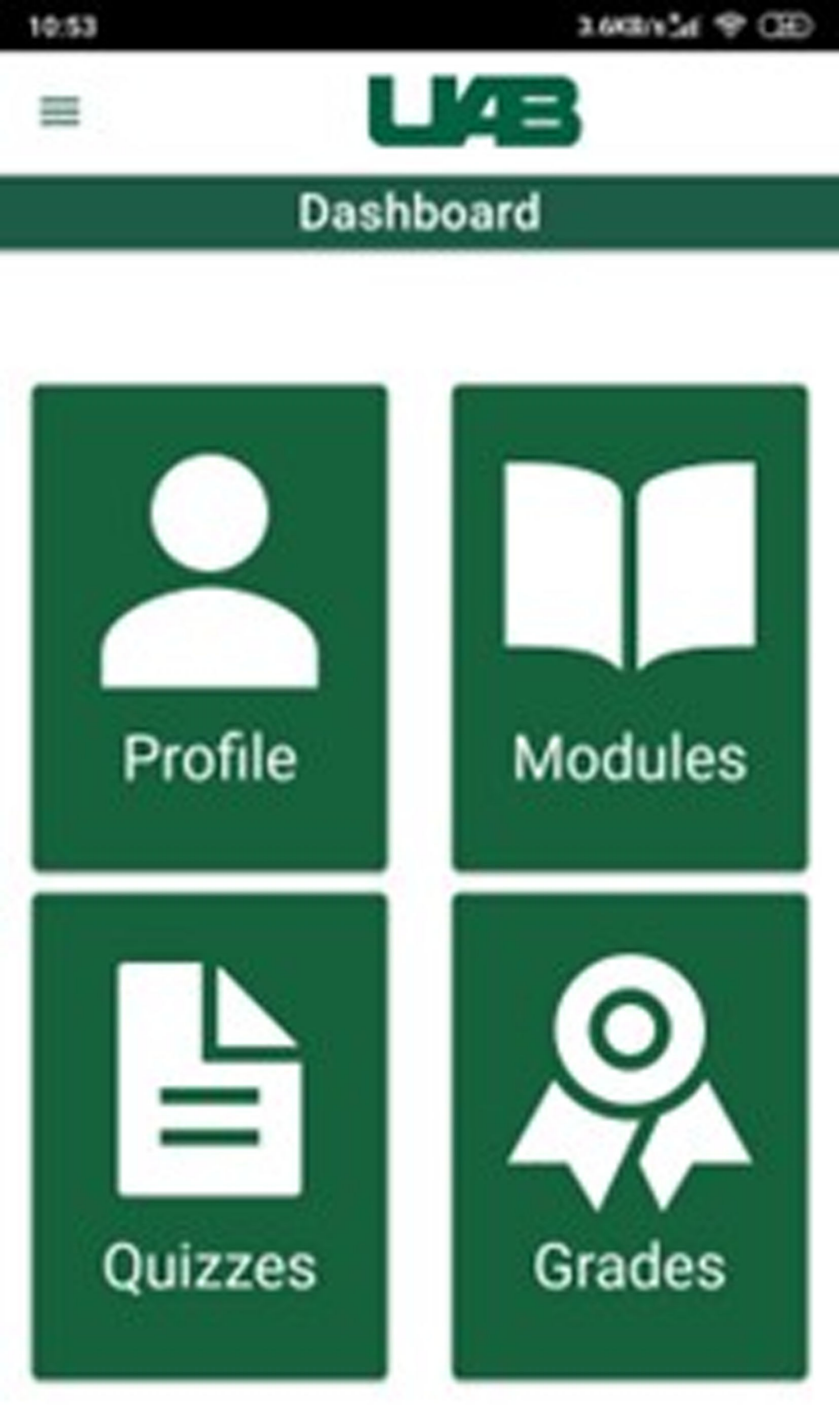
Screenshots of the cardiac implantable electronic device app.

We developed a de novo cloud-based system for the project. The CIED app (and the accompanying admin grading app) has 2 components: the mobile and web front-end, and the cloud-based back-end (Figure 1).

##### Front-end

The CIED app and the admin grading app are developed using Ionic (OutSystems), a cross-platform mobile app framework. This allows consistent functionality and user experience across iOS and Android devices. It also allows deploying a web app, which can be accessed from mobile phones or tablets as well as desktop web browsers.

##### Back-end

The back-end of this system is built using the Google Cloud. More specifically, we host the back-end computing, database, and analytics services based on the Google Firebase engine. We used the Google Cloud Firestore database to store the course modules, quiz questions, and per-user data such as grades. We used Firebase’s authentication engine to register and manage users.

### Analytics

For detailed user analytics, we used Google Firebase Analytics engine. It provided us with details on how the users interacted with the system and viewed different modules and videos. The per-user data on grades and quiz attempts provides us with information on the quiz attempts and performance. We collected pre and postlesson quizzes to compare performance before and after each lesson.

The evaluation of the educational material will occur in three phases. First, performance analytics will be performed, which will quantify the learning gain after compared to before taking the quizzes. Second, web analytics will determine the time taken by trainees in studying and answering the quizzes. Through this evaluation, the level of complexity of the material will be evaluated. Third, usability analytics will assess the learners’ satisfaction with the curricular content and app usability.

A total of 2 distinct applications were created; the User Study App, empowering cardiothoracic anesthesiology trainees to enhance their knowledge, and the Admin Grading App, streamlining quiz management and assessment. The successful integration of these apps creates a cohesive ecosystem that fosters growth, engagement, and continuous improvement within the anesthesiology community. Both apps are compatible with Android and iOS platforms.

### User Study App

This app is a knowledge hub catering to anesthesiology practitioners’ learning needs. It offers a collection of study materials, interactive resources, and self-assessment tools to foster professional growth. For usability testing, we are using the AttrakDiff test (https://www.attrakdiff.de/index-en.html). AttrakDiff is a standardized test designed to evaluate the user’s experience. It is widely used to assess the attractiveness of a product in terms of usability and appearance.

**Figure 2.**
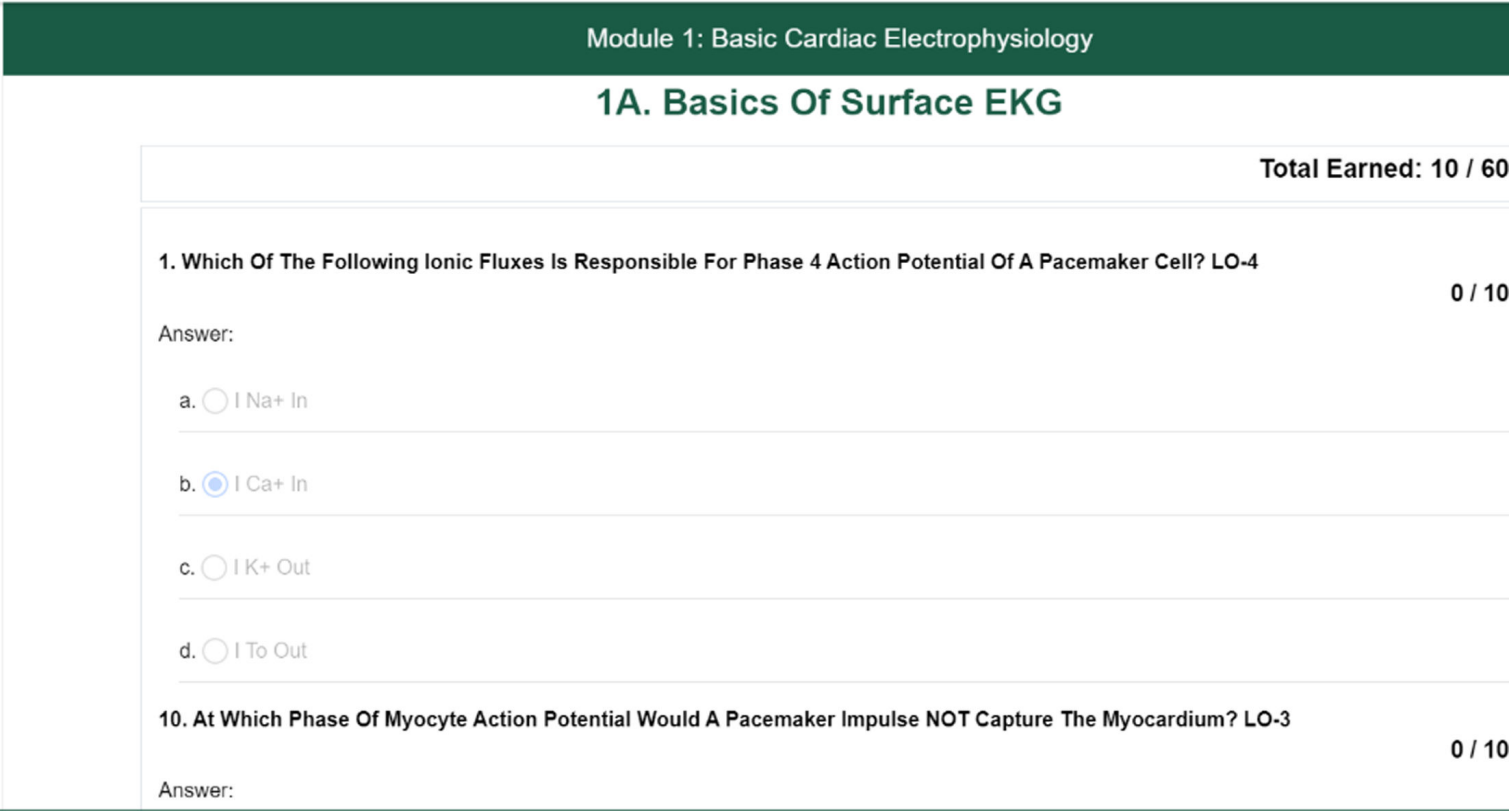
Screenshot of a modular instruction material-user app.

The User Study App is integrated with the prequiz condition (Figure 2).

#### Prequiz Requirement

Users must complete a prequiz assessment before gaining access to study materials.

#### Study Materials

**Figure 3.**
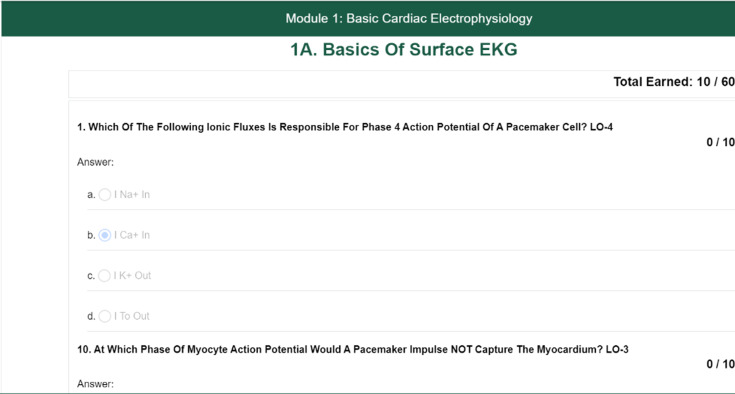
Screenshot of a quiz on a modular instruction material.

This includes a library of articles, multimedia content, and case studies tailored to various anesthesiology topics and expertise levels (Figure 3).

#### Interactive Quizzes

Quizzes were designed to complement the study materials and reinforce learning objectives.

#### Progress Tracking

Personalized user dashboards displaying prequiz scores and study progress, aiding in self-assessment and goal setting.

#### Learners’ Satisfaction Surveys

**Figure 4.**
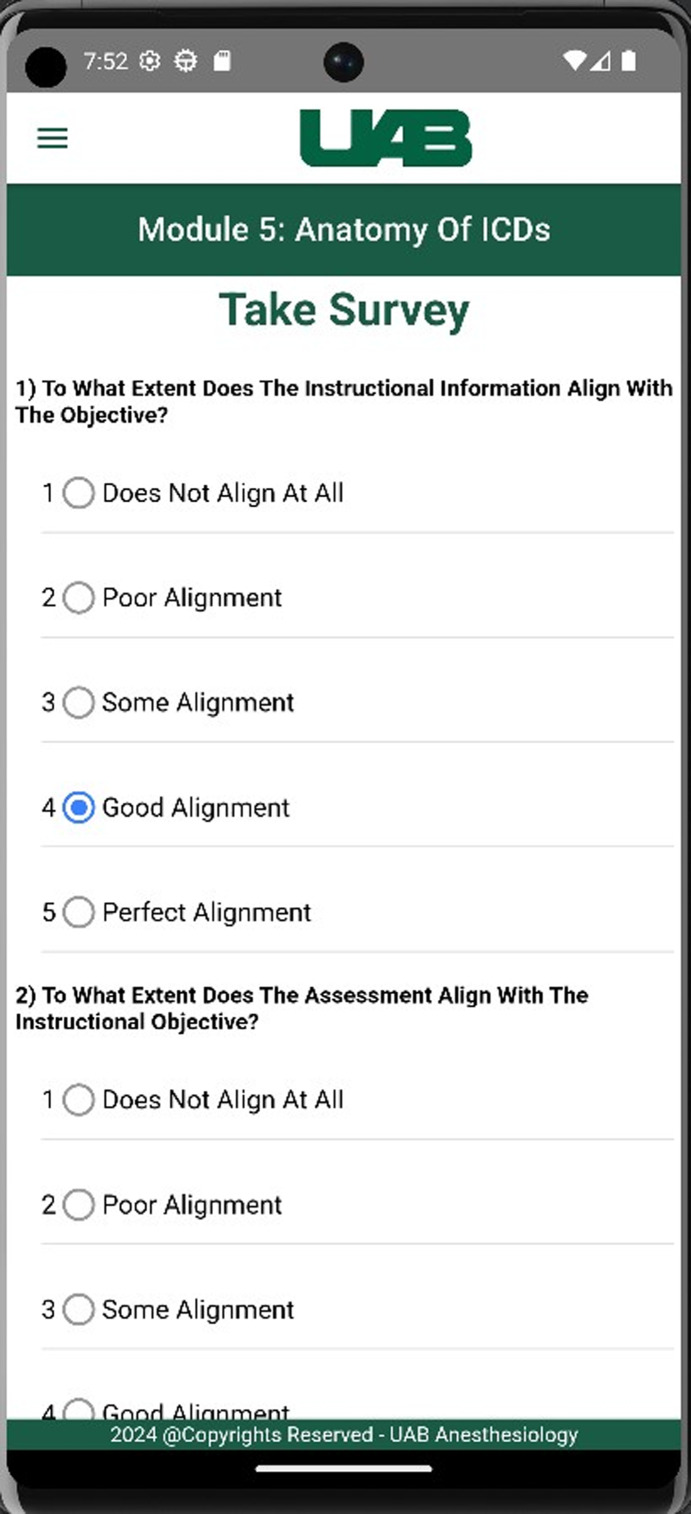
Screenshot of the Qualtrics survey embedded at the end of each instructional module.

Surveys on the learning experience of trainees were embedded at the end of each module. The surveys assess learners’ views on the clarity of the educational material, alignment of the educational objectives to goals and to the instructional and assessment material, what areas need further instruction and what technical issues arose during the navigating the module (Figure 4).

### Admin Grading App

The Admin Grading App equips administrators with powerful tools to manage quiz content and evaluate user performance. Key features are given below.

#### Admin Dashboard

This is a secure login portal providing access to quiz management, user profiles, and analytics.

#### Quiz Creation

This is a user-friendly interface allowing administrators to create and customize quizzes, tailoring them to specific learning objectives.

#### User Management

This includes comprehensive user profiles and progress reports, assisting administrators in understanding individual learning trajectories.

#### Grading Interface

**Figure 5.**
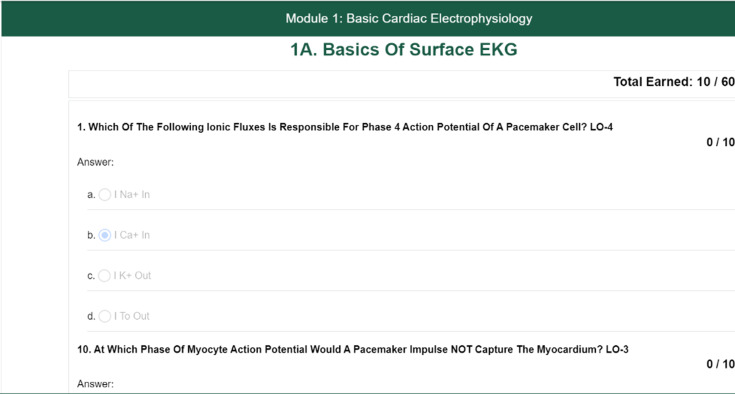
A screenshot of grading of a quiz-administrative app.

This was a grading system enabling assessment of quiz responses (Figure 5).

### Analytics and Reporting

Data visualization tools providing insights into overall user performance, popular study topics, and areas needing further emphasis.

### Integration and Security Measures: Fostering a Cohesive Ecosystem

The integration of the User Study and Admin Grading Apps is facilitated through shared databases, ensuring data synchronization and consistency. Security measures, including strong encryption and authentication protocols, safeguard sensitive user information and preserve the platform’s integrity.

## Discussion

### Principal Findings

This manuscript describes the process of designing, developing, and app delivering a comprehensive CIED curriculum for cardiothoracic anesthesiology trainees. This project has engaged an interdisciplinary team of cardiologists, electrophysiologists, technologists, computer scientists, and medical education. With the delivery of the curriculum, the investigators are in the process of collecting data on the platform implementation; user perceptions of usability, perceived effectiveness in promoting learning, and satisfaction with all components of the app; psychometrics of the learning assessments, and analysis of learning within each module.

While there are no previous studies on this subject to compare our work to, our mobile app goes in line with other studies that used mobile apps for educating anesthesiology trainees. Marty et al [29] designed a mobile app to facilitate programmatic assessment of anesthesiology trainees and tested its performance in five teaching institutions. The app provided insightful information about feedback completion times and documentation of learning goals. Monroe et al [30] implemented a mobile app for anesthesiology trainees rotating through a pediatric rotation in an academic institution. Similarly, Herbstreit et al [31] created a mobile platform formed of several department-specific apps and qualitatively demonstrated high satisfaction rates with the mobile learning content and pace. Compared with the above-mentioned studies, we have followed formal steps of creating a curriculum, consulted nationwide SMEs, and developed a curricular map that aligns instructional material and assessment to goals and objectives. Distinctively, our admin app uses web, performance, and usability analytics, which are either not covered or partially covered in other studies.

The project represents a collaborative effort between multiple teams to enhance trainees’ educational experience. As such, it extends the boundaries of cardiac anesthesiology, adding the new dimension of electrophysiology. Furthermore, this project represents a remarkable link between quality improvement, patient safety, and medical education. The effective implementation of our project is in line with similar improved outcomes with enhanced multidisciplinary team buildings and dynamics [32-34]. On the other hand, the program encountered some challenges before its successful implementation. First, the diversity in the learners’ training levels, including second-year rotating residents and cardiothoracic fellows, posed a challenge in the selection of instructional material. Second, the process of curricular instructional and assessment validation was more of a laborious process for SMEs due to the lengthiness of the material. Third, the choice of the instructional material was more of a challenge for basic learners given the density of the subject. This has led us to a multiple iterative process to adjust the curriculum to conform to the needs of a basic anesthesiology learner.

It is important to recognize some of the limitations of our project. At the time of writing of this manuscript, the project remains descriptive. We are currently collecting data on the learning gain and usability of the app. Therefore, the process of curricular refinement remains an iterative process. There is relatively a few number of SMEs recruited for the validation process of the curriculum. At the time of this recruitment, the curriculum remains in the initial stages and is subject to more iterations when more feedback is received from trainees. At such a point, more SMEs will be recruited on further refinements of the curriculum. Ideally, validation of curricular mapping should occur in a single step. In our case, given the lengthiness of the content, the process of curricular validation proceeded through validation of alignment of learning goals and objectives, followed by the instructional content and assessment validation. Furthermore, our SMEs were not confined to those with backgrounds in anesthesiology. While this may be regarded as a limitation, the diversity of SMEs’ backgrounds allows more robustness of the curriculum.

### Conclusions

The CIED app is a novel application-delivered curriculum on a complex and understudied subject in anesthesiology training. The curriculum adheres to conventional steps of curricular design. The m-delivery of the curriculum and the functionality of the app in the evaluation of the learners’ performance and satisfaction responds well to the challenges that face trainees in achieving synchronous learning and engages them in the learning and evaluation processes. As we continue to enhance and expand the CIED app, we envision its instrumental role in elevating the quality of anesthesiology practice worldwide, ultimately benefiting patient outcomes and health care excellence.

## Supplementary material

10.2196/60087 Multimedia Appendix 1 Rubric for assessment of psychomotor skills during device interrogation.

## Abbreviations

ACGME: Accredited Council of Graduate Medical Education
AVRNT: AV nodal reentrant tachycardia
CIED: cardiac implantable electronic devices
CRT: cardiac resynchronization therapy
ICD: implantable cardioverters defibrillators
m-learning: mobile learning
SME: subject matter experts
SVT: supraventricular tachycardia
VT: ventricular tachycardia
