# Supplementary material for: Cardiac Implantable Electronic Device Educational Application for Cardiac Anesthesiology Trainees: Tutorial on App Development
Source: JMIR Med Educ. 2025 Jul 29;11:e60087. doi: 10.2196/60087 (PMC12306915; doi:10.2196/60087)
Supplement: Multimedia Appendix 1 [file mededu-v11-e60087-s001.docx]

| **Appendix 1. Rubric for assessment of psychomotor skills during device interrogation** | | | | |
| --- | --- | --- | --- | --- |
|  | **Exceptional** | **Very good** | **Good** | **Poor** |
| Communication with instructor | explains the interrogation steps in an organized manner | Explains the majority of steps in an orderly way | Explains the majority of steps in unorderly way | Misses the communication of > 3 steps of interrogation and programming |
| 4-question device programming  (vendor, device, dependency, functionality) | Recognizes the 4 questions of device programming | Recognizes 3 of the 4 steps of device programming | Recognizes 2 of the 4 steps of device programming | Recognizes 1 of the 4 steps of device programming |
| 4-test (battery, capture, sensing, impedance testing) interrogation | Performs 4 tests of interrogation orderly | Performs 4 tests disorderly | Performs 3 of 4 tests | Performs < 3 of 4 tests |
| Programming based on surgical site | Appropriate independently | Appropriate with minimal instruction | Appropriate with substantial instruction | Fails to appropriately program according to site |
